# Adsorption of Co^2+^ and Cr^3+^ in Industrial Wastewater by Magnesium Silicate Nanomaterials

**DOI:** 10.3390/ma17091946

**Published:** 2024-04-23

**Authors:** Jing Bao, Yongjun Feng, Yong Pan, Juncheng Jiang

**Affiliations:** 1 Jiangsu Key Laboratory of Hazardous Chemicals Safety and Control, College of Safety Science and Engineering, Nanjing Tech University, Nanjing 210009, China; yongpannjut@163.com (Y.P.); junchengjiang@njtech.edu.cn (J.J.); 2 State Key Laboratory of Chemical Resource Engineering, Beijing University of Chemical Technology, No. 15 Beisanhuan East Road, Beijing 100029, China; yjfeng@mail.buct.edu.cn

**Keywords:** magnesium silicate nanomaterial, hierarchical pore structure, heavy metals Co^2+^ and Cr^3+^, adsorption, industrial wastewater

## Abstract

In this paper, two flower-like magnesium silicate nanomaterials were prepared. These and another two commercial magnesium silicate materials were characterized using a scanning electron microscope, the N_2_ adsorption–desorption method, and other methods. The structure–activity relationship between the adsorption performance of these four magnesium silicate materials and their specific surface area, pore size distribution, and pore structure was compared. The results showed that the 3-FMS modified by sodium dodecyl sulfonate (SDS) had the largest specific surface area and pore size, the best adsorption performance, and the largest experimental equilibrium adsorption capacity (*q_e,exp_*) for Co^2+^, reaching 190.01 mg/g, and Cr^3+^, reaching 208.89 mg/g. The adsorption behavior of the four materials for Co^2+^ and Cr^3+^ both fitted the pseudo-second-order kinetic model and Langmuir adsorption model, indicating that chemical monolayer uniform adsorption was the dominant adsorption process. Among them, the theoretical adsorption capacity (*q_m_*) of 3-FMS was the highest, reaching 207.62 mg/g for Co^2+^ and 230.85 mg/g for Cr^3+^. Through further research, it was found that the four materials mainly removed Co^2+^ and Cr^3+^ through electrostatic adsorption, surface metal ions (Mg^2+^), and acidic groups (-OH and -SO_3_H) exchanging with ions in solution. The adsorption performance of two self-made flower-like magnesium silicate materials for Co^2+^ and Cr^3+^ was superior to that of two commercial magnesium silicates.

## 1. Introduction

With the rapid development of global industry, the discharge of industrial wastewater containing heavy metal ions is also increasing year by year. Heavy metals are mostly non-degradable toxic substances that do not have natural purification capabilities. Once they enter the environment, it is difficult to remove them [1,2]. The harm inflicted by heavy metal pollution on the environment and human beings is attracting increasing attention [3].

Cobalt is widely used in industrial production and medical research. During the production and use of cobalt and its isotopes, a large amount of cobalt-containing wastewater is generated, which can have irreversible effects on the health of humans and animals. The corresponding hazards include general toxic effects, mutagenicity, carcinogenicity, and reproductive and developmental toxicity. Chromium pollution is mainly generated by the metallurgical industry, metal processing, electroplating, tanning, paint, printing and dyeing, the chemical industry, and other industries. Excessive intake of Cr^3+^ can cause diseases such as diabetes and arteriosclerosis and even cancer. In addition, Cr^3+^ can be transformed into more toxic Cr^6+^ under certain conditions, causing greater harm to human health [4,5,6,7]. Therefore, the treatment of wastewater containing Co^2+^ and Cr^3+^ is receiving a great deal of attention, and this article focuses on the removal of non-radiative Co^2+^ and Cr^3+^ from industrial wastewater. The commonly used removal methods currently include chemical precipitation, electrolysis, biological techniques, membrane separation, and adsorption [8,9,10,11,12,13]. Among them, adsorption is a commonly used method for removing heavy metals in water due to its simple operation, low relative cost, and low levels of secondary pollution [14,15].

Bin Bandar et al. [16] evaluated the ability of nanocellulose extracted from discarded palm leaves to remove Co^2+^ from industrial wastewater, and the maximum adsorption capacity of Co^2+^ at room temperature was 5.98 mg/g. Yuan et al. [17] successfully prepared a new class of glycine-derivative-functionalized metal organic frameworks (MIL-101-glycine, MIL-101-diaminoglycine, and MIL-101-triglycine) and used them to remove Co^2+^ from aqueous solutions. The results showed that the adsorption of MIL-101-triglyceride for Co^2+^ conformed to the Langmuir model, with a maximum saturated adsorption capacity of 232.6 mg/g. He et al. [18] studied the removal effect of plagioclase on Co^2+^ in aqueous solutions, and the results showed that the adsorption of Co^2+^ corresponded to a rapid pseudo-second-order kinetic process, which conformed to the Langmuir model. The maximum adsorption capacity of Co^2+^ at 35 °C was 8.88 mg/g. Lujaniene et al. [19] synthesized composite materials using chitosan, natural clay, montmorillonite, zeolite, crosslinking agents (epichlorohydrin, sodium tripolyphosphate, and glutaraldehyde), and plasticizers (glycerol) and studied the adsorption of composite materials with respect to Cs^+^, Co^2+^, and Eu^3+^ ions. The maximum adsorption capacities of composite materials for Cs^+^, Co^2+^, and Eu^3+^ were 1400 mg/g, 900 mg/g, and 18 mg/g, respectively. The experimental data better fit the Langmuir isotherm model, indicating that the composite materials exhibited good monolayer adsorption of Cs^+^, Co^2+^, and Eu^3+^ at uniform sites. The experimental data better fit the pseudo-second-order nonlinear kinetic model of most elements and adsorbents.

Guerue et al. [20] used low-cost natural diatomaceous earth to remove Cr^3+^ from water, using it in a continuous system at 30 °C for 80 min. The chromium removal rate in wastewater was 97%. Iddou et al. [21] studied the removal efficiency of Cr^3+^ from aqueous solutions using biological sludge waste activated sludge (WAS) from dairy filtration stations. The adsorption isotherm at pH 3 corresponded to the Langmuir model, with a maximum adsorption capacity of 25.64 mg/g. Mohan et al. [22] prepared low-cost activated carbon (ATFAC) using agricultural waste coconut shell fiber as a raw material and compared it with commercially available activated carbon fabric (ACF) to study the efficiency of their adsorption and purification processes for Cr^3+^ in tannery wastewater. The results showed that the maximum adsorption capacities of ATFAC and ACF at 25 °C were 12.2 and 39.56 mg/g, respectively. Kaur et al. [23] synthesized amino-functionalized mesoporous MCM-41 (NH2-MCM-41) and explored its use as an adsorbent for Cr^3+^ in water. The results showed that NH_2_-MCM-41 had a monolayer adsorption effect on Cr^3+^, with a maximum adsorption capacity of 83.33 mg/g. Gomez-Gonzalez et al. [24] prepared a high-capacity hybrid silica adsorbent using the sol–gel method, using an aqueous solution as a raw material and a sulfonic group as the chelating agent of the Cr^3+^ complex ion. The adsorption of Cr^3+^ was the highest at pH 3: 72.8 mg/g.

With the rapid development of molecular science, the physical properties of nanomaterials have been shown to be quite different from those of ordinary materials, with many new characteristics such as adsorption, catalysis, and molecular separation [25,26,27,28]. Nanomaterials are known as “one of the most important strategic high-tech materials in the 21st century”. In the past two decades, nanotechnology has been applied to almost all scientific and technological fields. When the particle size of a material is in the nanometer range, quantum effects begin to affect the properties and structure of the material, showing special physical and chemical properties, which can enhance various interfacial reactions [29,30,31], such as specific adsorption reactions for heavy metals. These factors play a significant role in the purification of heavy metal sewage, and there are already a large number of relevant research results. In recent years, magnesium silicate nanomaterials have shown great potential as emerging adsorbents for water treatment in the field of heavy metal removal. These materials number among the typical two-dimensional layered silicate compounds, which are abundant, cheap, easy to obtain, wear-resistant, and environmentally friendly and have strong thermal stability [32,33], a large surface area, and a rich pore structure. Their surfaces, interlayers, and inner walls contain a large number of silicon hydroxyl groups (Si-OH), which are easy to modify and have amphiphilic adsorption ability. They perform well in the adsorption of heavy metal cations in wastewater [34,35], but currently, there is little research on the adsorption of Co^2+^ and Cr^3+^ by magnesium silicate nanomaterials. Therefore, two common commercial magnesium silicate nanomaterials, namely, MP25 and JH713, and two laboratory-made flower-like magnesium silicate nanomaterials, namely, FMS and 3-FMS, were selected. This article will focus on the adsorption efficiency of these four nanometer magnesium silicate materials with respect to Co^2+^ and Cr^3+^ in wastewater.

## 2. Experimental Section

### 2.1. Main Reagents and Instruments

Both kinds of commercial magnesium silicate nanomaterials were purchased independently and in isolation, both of which are common products with a wide range of use at present. The product codes were MP25 and JH713, respectively. Both kinds of flower-like magnesium silicate nanomaterials were made by the laboratory [36]. The flower-like magnesium silicate nanomaterial without added sodium dodecyl sulfonate (SDS) was recorded as FMS, and the modified flower-like magnesium silicate nano material prepared by mixing 3% powder SDS (accounting for the mass fraction of the mixture) was recorded as 3-FMS.

Co(NO_3_)_2_·6H_2_O, AR, with purity greater than 99% was purchased from Shanghai Adamas Reagent Co., Ltd. (Shanghai, China); Cr(NO_3_)_3_·9H_2_O, AR, with purity greater than 99% was obtained from Shanghai Adamas Reagent Co., Ltd.; concentrated nitric acid, AR, with purity greater than 70% was obtained from Shanghai Aladdin reagent limited Company (Shanghai, China); and deionized water was made in our laboratory.

PHS 3C pH meter was obtained from Shanghai Lei Magnetic Instrument Factory (Shanghai, China); FA2004 electronic balance was obtained from Shanghai Ting Scale Co., Ltd. (Shanghai, China); TS-20H desktop constant temperature shaker was obtained from Shanghai Tiancheng Experimental Instrument Co., Ltd. (Shanghai, China); Shimadzu ICPS-7500 was obtained from Shimadzu Company, Kyoto, Japan; Zetas Izer Nano ZSE was obtained from Malven Panaco (Bournemouth, UK); and Bruker Vecor-22 Infrared Spectrometer was obtained from Bruker Instruments, Bremen, Germany.

### 2.2. Adsorption Kinetics Experiment

First, 49.39 mg of powdered Co (NO_3_)_2_·6H_2_O or 76.98 mg of powdered Cr (NO_3_)_3_·9H_2_O was dissolved in 100 mL of deionized water to make a Co^2+^ or Cr^3+^ solution (c.a. 100 mg/L), and the actual concentration was measured using a Shimadzu ICPS-7500 inductively coupled plasma atomic emission spectrometer (ICP-AES). Before measurement, the pH of the solution was adjusted to about 5.0 using HNO_3_ (the mass fraction of concentrated nitric acid is about 70%) to prevent hydrolysis.

A total of 50 mL of Co^2+^ or Cr^3+^ solution was added to a 100 mL conical flask, which was placed on a shaker (120 rpm) in a water bath at 25 °C; then, 20 mg of magnesium silicate sample was added into it, and timing was started. The liquid was sampled at different periods, and the sample was centrifuged at 3700 rpm for 5 min. Then, the supernatant was taken to measure the remaining Co^2+^ or Cr^3+^ and Mg^2+^ concentrations.

The amounts of Co^2+^ and Cr^3+^ adsorbed by the magnesium silicate nanomaterials were calculated using Equation (1)

(1) $$qt=\frac{\left(C_{0}-C_{t}\right)\times V}{m}$$

where *C*_0_ (mg/L) is the initial mass concentration of Co^2+^ and Cr^3+^, *C_t_* (mg/L) is the mass concentration of Co^2+^ and Cr^3+^ at time *t*, *V* (L) is the volume of Co^2+^ and Cr^3+^ solutions, *m* (g) denotes the mass of magnesium silicate nanomaterial, and *qt* (mg/g) denotes the corresponding amounts of Co^2+^ and of Mg^2+^ adsorbed by sample.

### 2.3. Adsorption Thermodynamics Experiment

A series of Co^2+^ and Cr^3+^ solutions with different mass concentrations (0~250 mg/L) were prepared. Here, 50 mL of Co^2+^ and Cr^3+^ solutions with different mass concentrations were added to 100 mL conical bottles, and about 20 mg of magnesium silicate material was added for adsorption experiments. A constant temperature of 25 °C together with shaker oscillation (120 r/min) were employed to allow absorbance for 12 h. A filtered syringe was used to extract the liquid, and ICP-AES was used to detect the ion concentrations of Co^2+^ and Mg^2+^, as well as Cr^3+^ and Mg^2+^, in the liquid. The adsorption of materials and the ion concentrations of Co^2+^ and Cr^3+^ were calculated using Formula (1).

## 3. Results and Discussions

### 3.1. Characterization of Magnesium Silicate Nanomaterials

**Figure 1:**
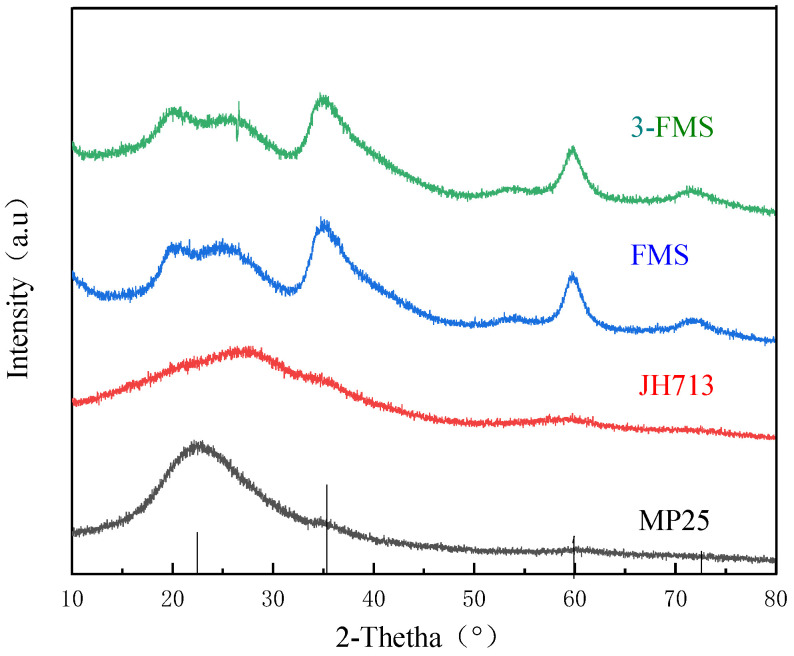
XRD spectrum of the four magnesium silicate materials.

Figure 1 shows the XRD patterns of the four magnesium silicate materials. It can be seen that the two commercial magnesium silicate materials are in an amorphous form; there is no obvious peak. The characteristic diffraction peaks (JCPDS No. 22-1155) [37] corresponding to the crystal faces of magnesium silicate [Mg_3_Si_2_O_5_(OH)_4_], namely, (002), (112), and (300), appear at 2θ = 12.45°, 35.90°, and 59.94°, respectively. The characteristic diffraction peaks of FMS and 3-FMS are the same, indicating that the addition of SDS did not change the crystal structure of the material, Moreover, the characteristic diffraction peaks of the three crystal planes—the half-diffraction angles—did not undergo significant shifts. According to the Bragg equation, the interplanar spacings *d* of the two self-made magnesium silicate materials before and after modification did not undergo significant changes. It can be speculated here that the appropriate amount of modifier SDS did not enter the interlayer of magnesium silicate but was loaded on its surface. This may be related to the crystal structure of magnesium silicate, which is similar to that of kaolinite [38]. There are no exchangeable cations in the interlayer, making it difficult for organic compounds to intercalate. Only a few strongly polar compound molecules, such as dimethylsulfoxide and acetic acid, can enter the interlayer [39,40]. Therefore, the modification preparation of SDS amounted to surface modification.

**Figure 2:**
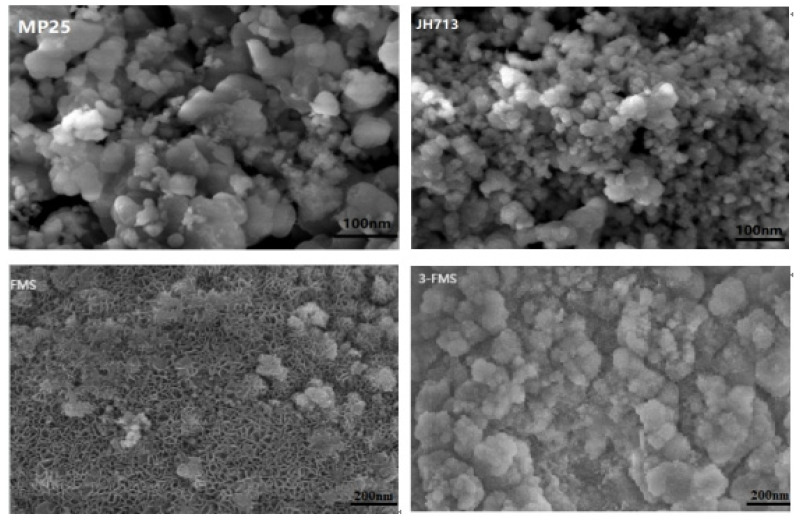
SEM diagram of the four magnesium silicate materials.

Figure 2 shows SEM images of the four magnesium silicate materials. The morphologies of both the MP25 and JH713 materials exhibit irregular nanoscale disordered structures, while both FMS and 3-FMS exhibit flower-like structures. When no SDS was added, the morphology of the FMS material exhibits a flower-like structure formed by many interlaced nanosheets, and the size of the FMS flower-like structure was measured using the Nano Measurer particle size analysis tool (Nano Measurer 1.2). The average diameter of the flower-like structure is approximately 200 nm. When 3 wt% SDS was added, 3-FMS retained an ordered flower-like structure, but its average diameter gradually increased to 280 nm. The average thickness of the nanosheets in the 3-FMS material was approximately 4.5 nm, and many large pore channels formed between these interlaced nanosheets, which facilitate the entrance and exit of substances; this result is consistent with the results of bulk density measurement: the bulk density of the FMS material is 0.85 g/cm^3^, while that of the 3-FMS material is 0.50 g/cm^3^, and the former bulk density is approximately twice that of the latter. Therefore, the addition of SDS made the flower-like magnesium silicate more porous, with a larger pore size and specific surface area, and the pore size distribution exhibited multilevel pores.

**Figure 3:**
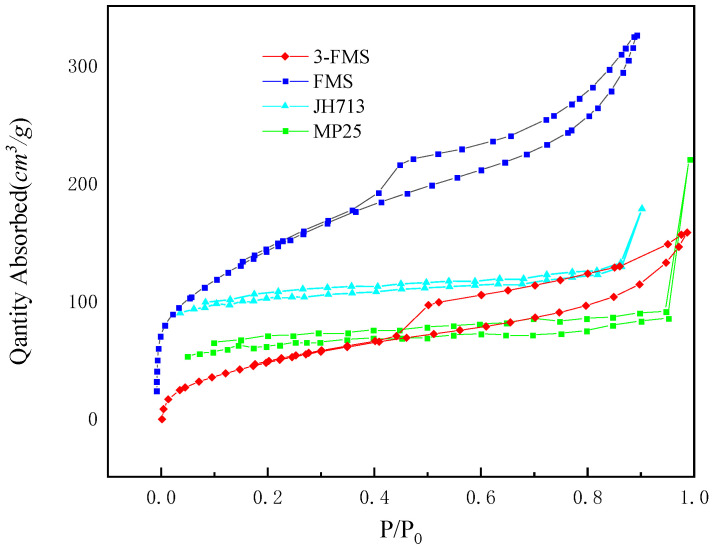
N_2_ adsorption-and-desorption curves of the four magnesium silicate materials.

Figure 3 shows the N_2_ adsorption–desorption curves and pore size distribution diagrams of the four magnesium silicate materials. It can be seen from the figure that the adsorption–desorption curves of the four magnesium silicate materials correspond to a type IV isotherm, with convexity in the later part of the curve and a typical H3 hysteresis loop in the middle section. The two commercial magnesium silicates have slit-shaped pores formed by layered structures, while the two self-made magnesium silicates have more obvious hysteresis loops than the two commercial magnesium silicates, indicating that they are composed of wedge-shaped pores formed by loosely layered structures [41].

**Figure 4:**
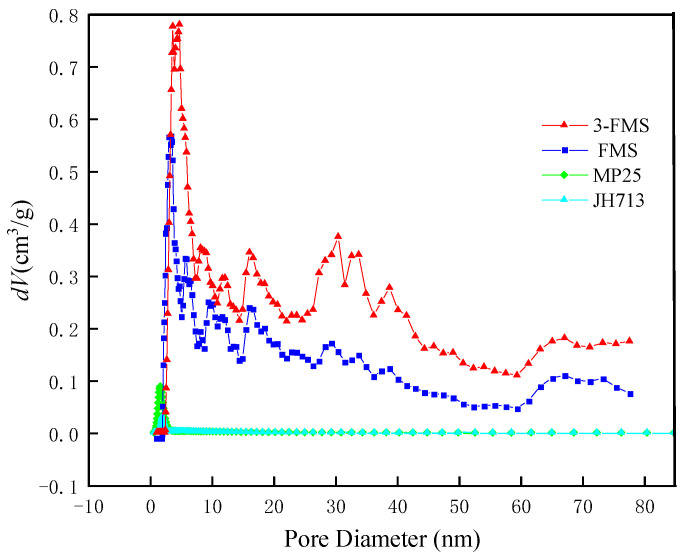
Pore size distribution of the four magnesium silicate materials.

Figure 4 shows the pore size distribution of the four magnesium silicate materials. It can be seen from the figure that the pore volume distributions of the two commercial magnesium silicates are similar, with pore sizes concentrated between 2 and 3 nm. The pore volume distributions of the two self-made magnesium silicate materials are also similar, with a similar pore volume distribution below 2 nm, showing a small number of micropores. The number of medium and large pores above 2 nm is significantly higher, and the number of 10~50 nm medium and large pores in 3-FMS is significantly higher than that in FMS.

Table 1 summarizes the BET data pertaining to the four materials. It can be seen that the average pore sizes of the two self-made magnesium silicate materials are larger than those of the two commercial magnesium silicates, and the specific surface area of JH713 is greater than that of FMS, but its average pore size is smaller than that of FMS, and its total pore volume is only slightly greater than that of FMS. At the same time, compared with FMS, with the addition of SDS, the specific surface area of 3-FMS increased significantly compared to that of FMS, reaching 502.42 m^2^/g, and the average pore size also increased from 4.329 nm to 7.28 nm. Therefore, the addition of SDS significantly increases the specific surface area and average pore size of self-made magnesium silicate materials.

**Figure 5:**
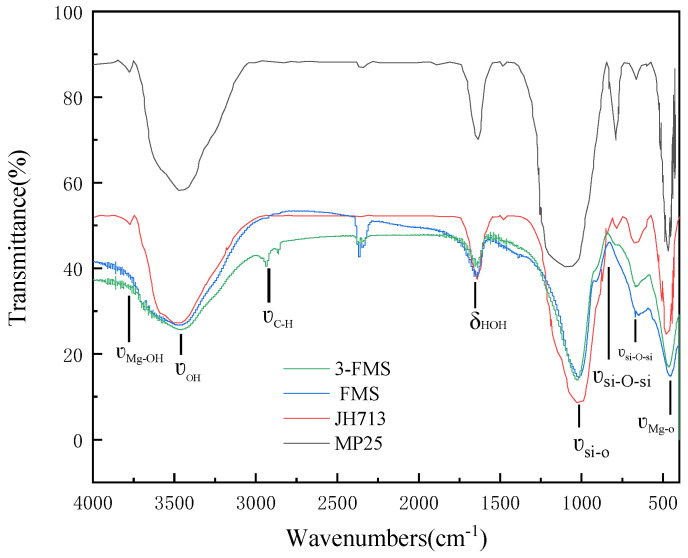
Infrared spectra of the four magnesium silicate materials.

The chemical bonds and functional groups contained in the four materials were characterized using a Vecor-22 infrared spectrometer (Bruker Vecor-22, Brooke Instruments, Dresden, Germany), and the corresponding infrared test diagram is shown in Figure 5.

The infrared spectra of the four magnesium silicate materials shows its characteristic infrared peaks: the adsorption peaks at 3680 cm^−1^ and 464 cm^−1^ correspond to the stretching vibration peaks of Mg-OH and Mg-O, respectively, while the peak at 663 cm^−1^ corresponds to the bending vibration peak of Si-O-Si. The stretching vibration peaks of Si-O-Si at 906 cm^−1^ and 1030 cm^−1^ correspond to the characteristic infrared peaks of magnesium silicate. The peaks at 1645 cm^−1^ and 3430 cm^−1^ correspond to the bending vibration peak of O-H in H-O-H. During the preparation of 3-FMS, SDS was added as a modifier. In addition to the above infrared characteristic peaks, there were also obvious stretching vibration peaks of C-H at 2960 cm^−1^ and 2870 cm^−1^. The characteristic peak of the sulfonic acid group (-SO_3_H) did not appear, probably due to the low amount added.

**Figure 6:**
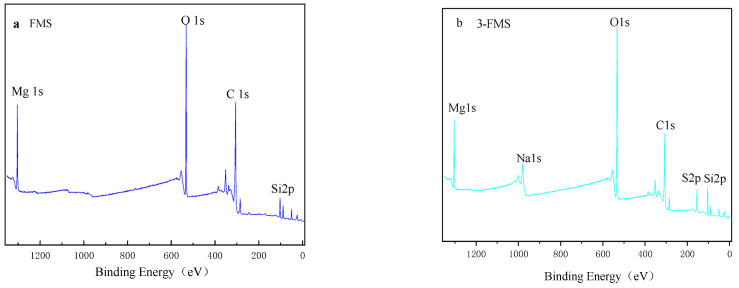
(**a**) XPS full spectrum of FMS; (**b**) XPS full spectrum of 3-FMS.

Using Thermo Scientific K-Alpha (Waltham, MA, USA), a surface elemental composition analysis was conducted on two self-made flower-like magnesium silicate samples. Figure 6a,b depict the full XPS spectra of the two flower-like magnesium silicates. As can be observed from the figure, the Mg, O, and Si peaks of the two materials have a Mg1s fitting peak binding energy of 1304.2 eV, and the four materials have an O1s fitting peak binding energy of 535.7 eV, which is between the SiO_2_ (533.3 eV) and MgO binding energies (531.8 eV). Therefore, these two peaks belong to the Mg-O-Si band of magnesium silicate, and the binding energy of the Si-OH bond in the two materials corresponds to Si^4+^ [42,43]. Two peaks of Na1s and S2p can also be observed in the XPS spectrum of 3-FMS, and their fitted peak binding energies are 1070.5 eV and 170.1 eV, indicating that sodium dodecyl sulfate successfully surface-modified magnesium silicate. The mass percentages of Mg, Si, O, Na, and S in the four materials are shown in Table 2. From the data in the table, it can be gleaned that the mass percentages of peaks with the same binding energy do not vary greatly, so their chemical states are basically unchanged.

### 3.2. Adsorption Properties of Magnesium Silicate Nanomaterials for Co^2+^ and Cr^3+^

#### 3.2.1. Adsorption Dynamics

The adsorption kinetics of the magnesium silicate nanomaterials mainly reflect their performance in terms of the rate of adsorption of the two metal cations and their adsorption capacity (equilibrium concentration).

**Figure 7:**
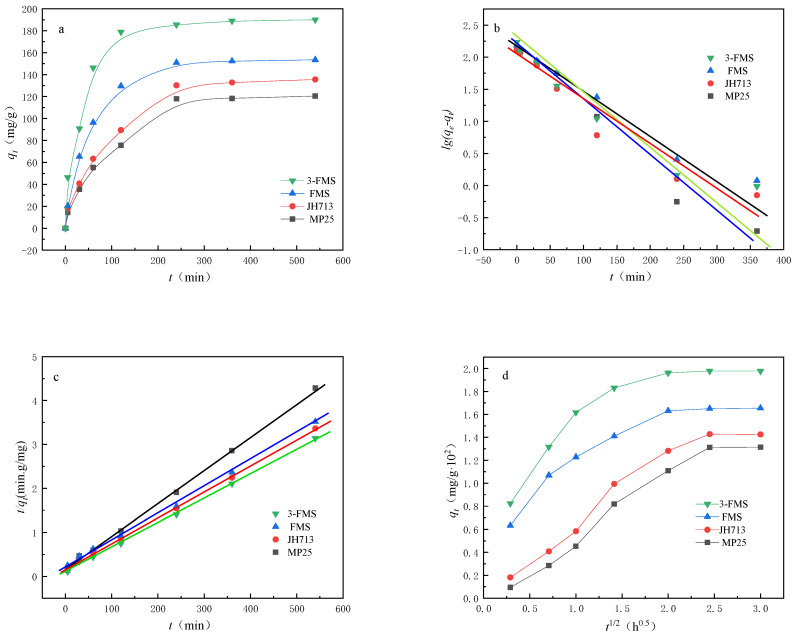
(**a**) The adsorption performance of the four materials for Co^2+^ over time, (**b**) linear fitting results of pseudo-first-order kinetics, (**c**) linear fitting results of pseudo-second-order kinetics, and (**d**) internal particle diffusion model.

**Figure 8:**
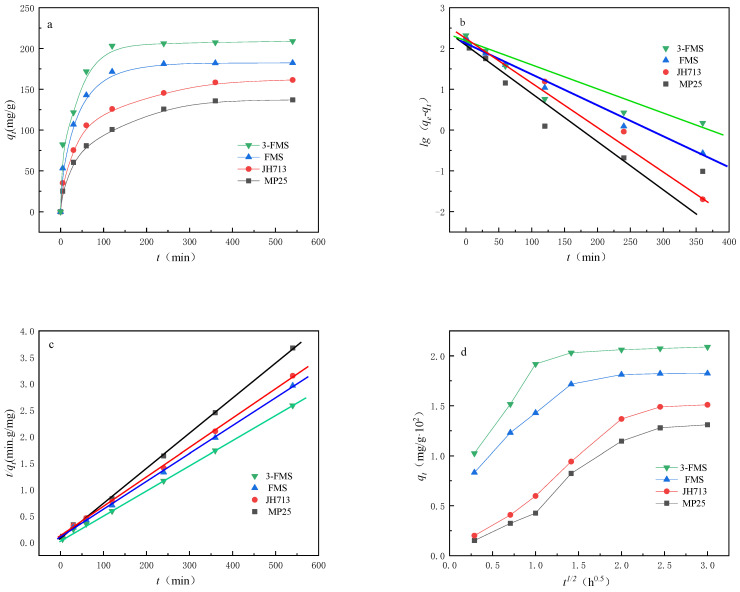
(**a**) The adsorption performance of the four materials for Cr^3+^ over time, (**b**) linear fitting results of pseudo-first-order kinetics, (**c**) linear fitting results of pseudo-second-order kinetics, and (**d**) internal particle diffusion model.

Figure 7 and Figure 8 show the changes in the adsorption performance of the four magnesium silicate materials for Co^2+^ and Cr^3+^ over time. As shown in Figure 7a and Figure 8a, the order of reaching adsorption equilibrium for the four materials is as follows: 3-FMS reaches adsorption equilibrium the fastest, followed by FMS, JH713, and MP25. The experimental equilibrium adsorption capacity (*q_e_*) also decreases in this order, with the experimental equilibrium adsorption capacity for Co^2+^ being 172.00, 153.49, 135.56, and 120.48 mg/g and the experimental equilibrium adsorption capacity for Cr^3+^ being 208.89, 182.41, 161.48, and 136.98 mg/g, respectively. This is because larger pore sizes are beneficial to mass transfer, which promotes the transmission and adsorption rate of Co^2+^ and Cr^3+^ in the pores of magnesium silicate, greatly shortening the equilibrium time and allowing them to reach adsorption equilibrium quickly. The larger specific surface area and wedge-shaped hole with openings at both ends provide more effective adsorption sites, allowing these materials to adsorb more Co^2+^ and Cr^3+^. Among the studied materials, 3-FMS reaches adsorption equilibrium faster than FMS, and it also has a larger experimental equilibrium adsorption capacity (*q_e_*), which is due to the addition of the modifier SDS, which increases the specific surface area of 3-FMS and results in a more pronounced hierarchical pore structure compared to FMS.

To further investigate the adsorption behavior of Co^2+^ and Cr^3+^ for four magnesium silicate nanomaterials, we used three typical adsorption kinetic models, namely, a pseudo-first-order kinetic model (Equation (2)), a pseudo-second-order kinetic model (Equation (3)), and a diffusion kinetic model, that is, the Weber–Morris intraparticle diffusion model (Equation (4)) [44,45,46], combined with kinetic theory to study adsorption behavior.

(2) $$\log\left(q_{e}-q_{t}\right)=\log\left(q_{e}\right)-\frac{k_{1}}{2.303}t$$

(3) $$\frac{t}{q_{t}}=\frac{1}{k_{2}q_{e}^{2}}+\frac{t}{q_{e}}$$

(4) $$q_{t}=k_{dif}\sqrt{t}+C$$

where *q_e_* (mg/g) and *q_t_* (mg/g) are the adsorption amounts of Co^2+^ and Cr^3+^ at equilibrium and time *t* (min), respectively; *k*_1_ (1/min), *k*_2_ (g/mg/min), and *k_dif_* (mg/g/h^1/2^) are the rate constants of the pseudo-first-order kinetic model, the pseudo-second-order kinetic model, and the interparticle diffusion model, respectively; and *C* is the intercept.

The parameters *k*_1_, *k*_2_, and *q_e_* were determined through linear relationships, as shown in Figure 7 and Figure 8, and Table 3 and Table 4. From the graphs, it can be seen that the adsorption behavior of the four materials for Co^2+^ and Cr^3+^ is more consistent with a pseudo-second-order adsorption kinetic model, with R^2^ values closer to 1, and the calculated values of *q_e_* are also consistent with the measured values. This indicates that chemisorption plays an important role in the adsorption of Co^2+^ and Cr^3+^ on nanoscale magnesium silicate materials [47].

The adsorption process generally consists of the following three steps [48]: the rapid external diffusion phase, consisting of the diffusion of the adsorbate to the boundary layer of the adsorbent or adsorption on the outer surface; the intraparticle diffusion phase, constituting the mass transfer stage of the diffusion of the adsorbate to the interior of the particle (in the pore channel); and the slow equilibrium phase. As can be seen from Figure 7d and Figure 8d, the two commercial magnesium silicate materials are clearer than the two self-made magnesium silicate materials in the three steps of the figure because they have a large number of micropores smaller than 2 nm. The transport speed of adsorbate in micropores is slower than that in mesopores and macropores, so the processes of diffusion on the outer surface layer of the adsorbent and mass transfer to the interior of adsorbent particles are longer, resulting in a slower adsorption equilibrium stage. However, the pore distribution of the two self-made magnesium silicate materials is dominated by mesopores and macropores, with almost no micropores smaller than 2 nm, which enables rapid completion of the second stage of adsorbent particle internal diffusion. The second stage and the first stage are almost merged into one, so only two steps of diffusion can be seen in the figure. Upon comparing the diffusion behavior of the two self-made magnesium silicate materials, it can be seen from the figure that the diffusion rate and equilibrium adsorption capacity of 3-FMS are higher than those of FMS, because 3-FMS has a multilevel pore structure with more mesopores and macropores than FMS. It was inferred that the addition of modifier SDS further improves the pore size distribution of flower-like magnesium silicate materials and enhances their adsorption capacity for Co^2+^ and Cr^3+^.

#### 3.2.2. Adsorption Thermodynamics

The adsorption thermodynamics of magnesium silicate nanomaterials mainly reflect their ability to adsorb two metal cations and further reveal their adsorption mechanisms, providing theoretical guidance for the development of new magnesium silicate adsorption nanomaterials.

**Figure 9:**
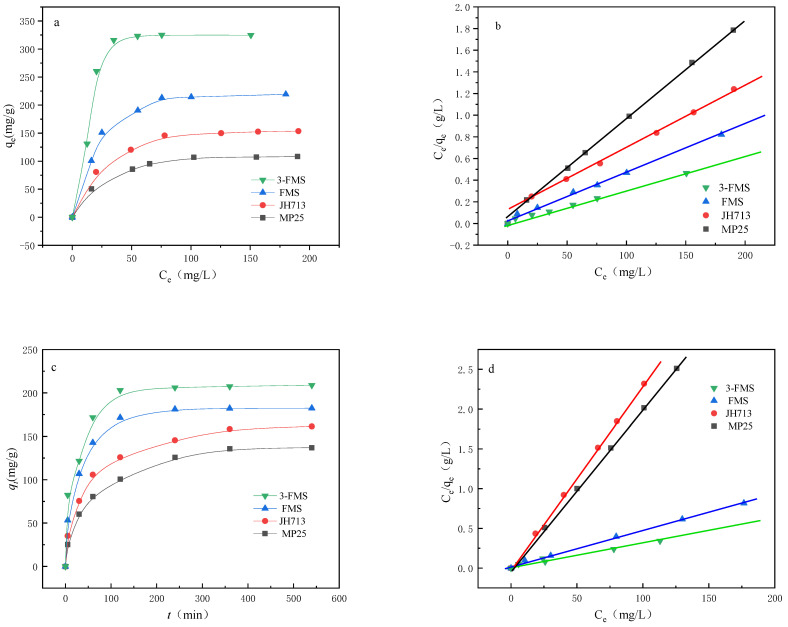
(**a**) The adsorption isotherm for the adsorption of Co^2+^ by four materials at pH ≈ 5.0. (**b**) Linear fitting of Langmuir model of Co^2+^ with respect to the four materials. (**c**) The adsorption isotherm of Cr^3+^ by four materials at pH ≈ 5.0. (**d**) Linear fitting of Langmuir model of Cr^3+^ with respect to the four materials.

Figure 9a,c show the adsorption isotherms of the four materials for Co^2+^ and Cr^3+^ in water, respectively, and linear fitting was performed using the Langmuir adsorption model (Equation (5)). The fitting results are shown in Figure 9b,d and Table 5 and Table 6.

(5) $$\frac{C_{e}}{q_{e}}=\frac{1}{k_{L}q_{m}}+\frac{C_{e}}{q_{m}}$$

where *q_e_* (mg/g) and *q_m_* (mg/g) are the equilibrium adsorption capacity and the maximum adsorption capacity, respectively; *C_e_* is the equilibrium concentration of Co^2+^ and Cr^3+^ in the solution; *K_L_* is the Langmuir equilibrium constant. *q_m_* and *K_L_* were obtained by fitting the data with a linear relationship between *C_e_/q_e_* and *C_e_*.

It can be seen that the four materials exhibited good adsorption of Co^2+^ and Cr^3+^, which is in line with the Langmuir adsorption model, indicating that they can all carry out uniform adsorption of single molecular layers. By comparing the theoretical maximum adsorption (*q_m_*) in Table 5 and Table 6, it can be seen that the raking of the theoretical maximum adsorption (*q_m_*) of the four materials for Co^2+^ and Cr^3+^ from low to high MP25, JH713, FMS, and 3-FMS, indicating conformity with the experimental equilibrium adsorption trend measured above.

Comparing the fitting data for the two self-made flower-like magnesium silicates shows that with the addition of SDS, the *q_m_* value of the resulting material increases, and the theoretical maximum adsorption capacities of 3-FMS for Co^2+^ and Cr^3+^ are 207.6 and 226.1 mg/g, respectively, higher than the results reported in Table 7. This indicates that the 3-FMS material prepared via SDS modification is an efficient adsorbent with excellent performance.

### 3.3. Adsorption Mechanism of Heavy Metals Co^2+^ and Cr^3+^

Adsorption is an interfacial phenomenon that is mainly caused by electrostatic adsorption, van der Waals forces, chemical bonds, etc., between an adsorbent and an adsorbate. According to the binding force between the adsorbent and adsorbate, adsorption can be divided into physical adsorption and chemical adsorption. The adsorption force of physical adsorption is generated by electrostatic adsorption and van der Waals forces, which are generally reversible. Physical adsorption can be either single-layer adsorption or multi-layer adsorption. The adsorption force of chemical adsorption is generated by chemical bonds, mostly constituting single-layer selective adsorption. It should be noted that there is no clear distinction between the two types of adsorption in the adsorption process, and physical adsorption and chemical adsorption often occur simultaneously.

**Figure 10:**
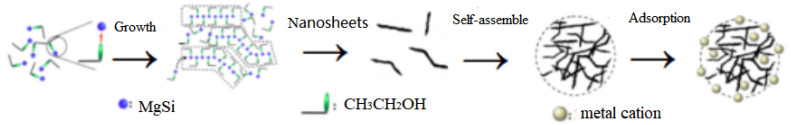
Schematic diagram of the growth and adsorption process of FMS.

According to the results regarding adsorption thermodynamics and kinetics, the adsorption behaviors of the four materials all conform to the pseudo-second-order adsorption kinetic model and Langmuir adsorption model, indicating that single-molecule layer chemisorption plays a dominant role in the entire adsorption process. At the same time, when the pH ≈ 5, the surfaces of the four magnesium silicate nanomaterials are all negatively charged, so they also adsorb positively charged Co^2+^ and Cr^3+^ through electrostatic adsorption or physical adsorption. Figure 10 is a schematic diagram of the growth and adsorption process of FMS [49].

#### 3.3.1. Ion Exchange

According to the adsorption kinetics analysis above, the adsorption behavior of the four magnesium silicate materials for Co^2+^ and Cr^3+^ is more consistent with the pseudo-second-order adsorption kinetic model, indicating that chemical adsorption plays an important role in the adsorption of Co^2+^ and Cr^3+^ on nanometer-sized magnesium silicate materials. Through analyzing the adsorption experiment data for Mg^2+^ and Co^2+^, Mg^2+^ and Cr^3+^ in the clear solution, it can be seen that the amount of Mg^2+^ in the clear solution increases with the decrease in Co^2+^ or Cr^3+^, which indicates that the type of chemical adsorption here may be the exchange of Mg^2+^ bound to the surface of the material by ionic bonds with solute ions in the solution; that is, the adsorption mechanism of the modified flower-like magnesium silicate adsorption material for Co^2+^ or Cr^3+^ is an exchange reaction between Mg^2+^ and Co^2+^ or Cr^3+^, i.e., solidification adsorption. The schematic expression is shown in Equations (6) and (7).

Mg--R + Co^2+^ === Mg^2+^ + Co (6)

3Mg--R + 2Cr^3+^ === 3Mg^2+^ + Cr_2_R_3_ (7)

R represents the rest of the chemical formulae for magnesium silicate.

Secondly, the four magnesium silicate materials contain large quantities of active hydroxyl groups (-OH) in their structures, which can also undergo ion exchange reactions with Co^2+^ or Cr^3+^ in aqueous solutions, as shown schematically in Equations (8) and (9).

2◉-OH + Co^2+^ === Co (◉-O)_2_ + 2H^+^ (8)

3◉-OH + Cr^3+^ === Cr (◉-O)_3_ + 3H^+^ (9)

◉ represents the other parts of the chemical molecular formula of magnesium silicate.

Finally, the structure of the modified magnesium silicate material 3-FMS also contains the acidic group-SO_3_H, which also undergoes an ion exchange reaction with Co^2+^ or Cr^3+^ in an aqueous solution, as shown schematically in Equations (10) and (11).

2✷-SO_3_H + Co^2+^ === Co (✷-SO_3_)_2_ + 2H^+^ (10)

3✷-SO_3_H + 2Cr^3+^ === Cr_2_ (✷-SO_3_)_3_ + 3H^+^ (11)

✷ represents other parts of SDS.

#### 3.3.2. Electrostatic Adsorption

**Figure 11:**
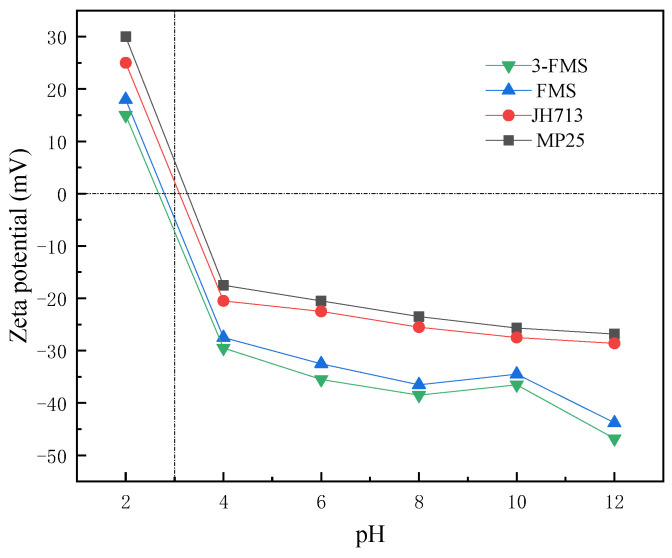
Zeta potentials of the four materials at different pH levels.

Magnesium silicate has a two-dimensional layered structure. It has a large number of silicon hydroxyl groups (Si-OH) on its surface, and Si-OH adsorbs or dissociates hydrogen ions on this surface, endowing all four magnesium silicate materials with positive, negative, or electroneutral surfaces. Figure 11 shows the Zeta potential test results for each flower-like magnesium silicate material, and their IEP values were measured between 2.5 and 3.5. Therefore, when the pH is about 5, the surface of each material is negatively charged; thus, the four kinds of nano-magnesium silicate will also absorb some positively charged Co^2+^ and Cr^3+^ ions through electrostatic electricity. It can be seen from the figure that the Zeta negative potential of the two self-made magnesium silicate materials is significantly lower than that of the two commercial magnesium silicate materials; therefore, the electrostatic adsorption effect on the two metal cations is stronger.

Slightly different from FMS, the 3-FMS adsorbent material was prepared by adding SDS as a modifier. SDS contains sulfonic acid (-SO_3_H), which dissociates in water and generates hydrophilic anions [50,51]. This characteristic endows the surface of 3-FMS with a negative charge, thereby promoting its adsorption of Co^2+^ and Cr^3+^ in water. In addition, a large number of methyl and ethyl groups contained in SDS can easily be wrapped on the particle surface to form micelles, which strengthens the electrostatic attraction with Co^2+^ and Cr^3+^ in the adsorbate and improves the adsorption efficiency of nano adsorption materials. At the same time, SDS easily forms a surface facial mask on the particle surface, which can prevent the agglomeration of nano particles in water. Therefore, the Zeta negative potential value of 3-FMS is lower than that of FMS, and its electrostatic adsorption effect on the two metal cations is stronger than that of FMS.

Although the number of magnesium ions in wastewater increases after adsorption, magnesium is a necessary element of the human body. Its allowable content in water is high, and this increase is far less than the maximum concentration limit of magnesium ions in drinking water. Therefore, magnesium silicate is a safe absorbent.

The results obtained from the above series of experimental data analyses are consistent with the theoretical speculation. The adsorption efficiency of the two self-made flower-like nano magnesium silicate materials for Co^2+^ or Cr^3+^ in industrial wastewater is better than that of the two commercial nano magnesium silicate materials. In the self-made magnesium silicate materials, 3-FMS is better for Co^2+^ or Cr^3+^ in industrial wastewater. Its adsorption efficiency is better than that of FMS, so sodium dodecyl sulfonate is an effective modifier for self-made flower-like magnesium silicate materials.

## 4. Conclusions

Through experimental comparison, the adsorption behaviors of the four magnesium silicate nano materials with respect to Co^2+^ and Cr^3+^, their adsorption efficiency, and the structure–effect relationship between specific surface area and pore structure were studied. The results show that the adsorption efficiency of the two kinds of self-made magnesium silicate is better than that of the two kinds of commercial magnesium silicate materials. The self-made magnesium silicate materials show an obvious multi-stage pore structure. Their pore distribution is mainly concentrated in the medium and large pore ranges of 2~50 nm and 50~80 nm. The specific surface area of 3-FMS prepared via SDS modification is larger. The distribution of pores in its structure is better than that of FMS. Having a large number of medium and large pores is conducive to the rapid transmission of an absorbent in the pores, and a multi-stage pore structure is conducive to increasing the utilization rate of holes. These qualities not only greatly accelerate the adsorption of Co^2+^ and Cr^3+^ but also significantly improve the equilibrium adsorption of Co^2+^ and Cr^3+^, amounting to up to 172.00 mg/g and 208.89 mg/g, respectively. In short, the adsorption efficiency of the two self-made magnesium silicate materials is better than that of the two commercial magnesium silicate materials. The adsorption behavior of the four magnesium silicate nano materials with respect to Co^2+^ and Cr^3+^ conforms to the quasi-secondary adsorption kinetics model, and their thermodynamic behavior conforms to the Langmuir adsorption model, indicating that the chemical adsorption of a single molecular layer plays an important role in the adsorption process of two metal cations by a series of materials; four nano silicon magnesium acid materials achieve the purpose of removing Co^2+^ or Cr^3+^ from industrial wastewater through ion exchange and electrostatic adsorption.

## Figures and Tables

**Table 1:** BET characterization results for four magnesium silicate materials.

Sample	Surface Area (m^2^/g)	Average Pore Diameter (nm)	Pore Volume (cm^3^/g)
MP25	230.59	2.42	0.14
FMS	241.75	4.33	0.26
JH713	367.45	3.01	0.27
3-FMS	502.42	7.28	0.63

**Table 2:** Mass percentages of elements contained in two magnesium silicate materials.

Material	O (%)	Mg (%)	Si (%)	S (%)	Na (%)	C (%)
FMS	52.12	18.37	17.04	/	/	12.46
3-FMS	51.11	17.86	16.57	0.61	0.36	13.50

**Table 3:** Fitting values of Co^2+^ adsorption kinetics of the four magnesium silicate materials.

Material	*q*_e,exp_ (mg/g)	Pseudo-First-Order Model			Pseudo-Second-Order Model		
		*q*_e,cal_ (mg·g^−1^)	*k*_1_ (min^−1^)	*R* ^2^	*q*_e,cal_ (mg·g^−1^)	*k*_2_(g·mg^−1^·min^−1^)	*R* ^2^
MP25	120.48	107.73	0.27	0.941	119.80	0.53	0.999
JH713	135.56	110.45	0.32	0.935	137.56	0.65	0.999
FMS	153.48	113.56	0.47	0.952	159.05	0.81	0.999
3-FMS	190.01	172.87	0.69	0.956	195.38	1.02	0.998

**Table 4:** Fitting values of Cr^3+^ adsorption kinetics of the four magnesium silicate materials.

Material	*q*_e,exp_ (mg/g)	Pseudo-First-Order Model			Pseudo-Second-Order Model		
		*q*_e,cal_ (mg·g^−1^)	*k*_1_ (min^−1^)	*R* ^2^	*q*_e,cal_ (mg·g^−1^)	*k*_2_ (g·mg^−1^·min^−1^)	*R* ^2^
MP25	136.98	100.84	0.54	0.936	135.73	0.14	0.998
JH713	161.48	145.54	0.33	0.973	163.25	0.76	0.999
FMS	182.40	150.79	0.47	0.945	190.45	0.83	0.998
3-FMS	208.89	169.89	0.61	0.972	220.63	1.04	0.999

**Table 5:** Langmuir fitting parameters of material adsorption of Co^2+^.

Material	*K_L_* (L/mg)	*q_m_* (mg/g)	*R* ^2^
MP25	0.56	150.85	0.999
JH713	0.69	162.52	0.999
FMS	0.58	185.14.	0.999
3-FMS	1.07	207.62	0.999

**Table 6:** Langmuir fitting parameters of material adsorption of Cr^3+^.

Material	*K_L_* (L/mg)	*q_m_* (mg/g)	*R* ^2^
MP25	0.47	162.43	0.999
JH713	0.74	180.72	0.999
FMS	0.53	200.71	0.999
3-FMS	1.03	230.85	0.999

**Table 7:** Maximum adsorption capacities(*q_m_*) for adsorption of Co^2+^ and Cr^3+^ onto various adsorbents.

Materials	*q_m_* (mg·g^−1^)	
	Co^2+^	Cr^3+^
Nanocellulose [16]	5.98	/
Plagioclase [18]	8.88	/
WAS [21]	/	25.64
ATFAC [22]	/	12.21
ACF [22]	/	39.56
NH2-MCM-41 [23]	/	83.33

## Data Availability

Data are contained within the article.
